# Simulating and optimizing compound refractive lens-based X-ray microscopes

**DOI:** 10.1107/S160057751602049X

**Published:** 2017-02-20

**Authors:** Hugh Simons, Sonja Rosenlund Ahl, Henning Friis Poulsen, Carsten Detlefs

**Affiliations:** a Department of Physics, Technical University of Denmark, 2800 Kgs Lyngby, Denmark; b European Synchrotron Radiation Facility, 71 Avenue des Martyrs, 38000 Grenoble, France

**Keywords:** X-ray imaging, X-ray microscopy, X-ray optics, compound refractive lens, ray transfer matrix

## Abstract

A comprehensive optical description of compound refractive lenses (CRLs) in condensing and full-field X-ray microscopy applications is presented. The formalism extends ray-transfer matrix analysis by accounting for X-ray attenuation by the lens material. Closed analytical expressions for critical imaging parameters such as numerical aperture, spatial acceptance (vignetting), chromatic aberration and focal length are provided for both thin- and thick-lens imaging geometries. These expressions show that the numerical aperture will be maximized and chromatic aberration will be minimized at the thick-lens limit. This limit may be satisfied by a range of CRL geometries, suggesting alternative approaches to improving the resolution and efficiency of CRLs and X-ray microscopes.

## Abstract 2

A comprehensive ray-transfer matrix formalism for the use and design of compound refractive lens-based X-ray microscopes is presented, including closed analytical expressions for key optical parameters. These form the basis of an optimization, which reveals that thick-lens imaging geometries are ideal in terms of spatial resolution and achromaticity.

## 1. Introduction   

Many recent advances in synchrotron X-ray imaging can be attributed to X-ray focusing optics (Ice *et al.*, 2011 ▸). These optics may operate *via* three possible principles: (i) diffraction, such as in Fresnel zone plates (Kirz, 1974 ▸) and multilayer Laue lenses (Kang *et al.*, 2006 ▸); (ii) total reflection, such as in Kirkpatrick–Baez (Kirkpatrick & Baez, 1948 ▸) and Wolter (Wolter, 1952 ▸) mirrors, and lobster-eye (Inneman *et al.*, 1999 ▸) and Kumakhov (Kumakhov & Komarov, 1990 ▸) lenses; and (iii) refraction, such as in prisms (Cederstrom *et al.*, 2000 ▸) and compound refractive lenses (CRLs) (Snigirev *et al.*, 1996 ▸). In the hard X-ray regime (*E* > 15 keV), CRLs (linear arrays of refractive lenslets) are widely used due to their relatively low cost, ease-of-use and efficiency. Furthermore, their focal length can be actively varied by adjusting the number of lenslets (Vaughan *et al.*, 2011 ▸). However, the spatial resolution of CRL-based imaging systems is typically 50–100 nm (Schroer *et al.*, 2005 ▸), worse than that of other optics at comparable energies: 7 nm (Yamauchi *et al.*, 2011 ▸), 8 nm (Morgan *et al.*, 2015 ▸) and 20 nm (Vila-Comamala *et al.*, 2012 ▸) have been reported from microscopes based on mirrors, multilayer Laue lenses and Fresnel zone plates, respectively. Nonetheless, the advantages of CRLs make them uniquely suitable for *in situ* experiments where efficiency, large working distances and high X-ray energies are required. Under such circumstances, improving the spatial resolution of CRLs could facilitate new opportunities for multi-scale characterization.

One route to improving spatial resolution is by optimizing the CRL geometry (Chen *et al.*, 2014 ▸). Numerical optimization requires concise analytical expressions for parameters such as focal length, transmission and aberration. Furthermore, these expressions are essential for imaging techniques that involve sampling data in grids such as ptychography (Schroer *et al.*, 2008 ▸), scanning X-ray microscopy (Schroer *et al.*, 2005 ▸) or dark-field X-ray microscopy (Simons *et al.*, 2015 ▸). The optical theory of CRLs and CRL-based imaging systems has been addressed by various approaches such as ray-transfer matrices (RTMs) (Protopopov & Valiev, 1998 ▸; Pantell *et al.*, 2003 ▸) [including Gaussian beam variants (Poulsen & Poulsen, 2014 ▸)], Monte Carlo ray tracing (Sanchez~del Rio & Alianelli, 2012 ▸), wavefront propagation methods (Kohn, 2003 ▸) and others (Lengeler *et al.*, 1999 ▸). While these have greatly furthered the design and implementation of CRLs, no single formalism fulfills the core requirements for optimization: (i) simple, closed expressions, (ii) broad applicability to both condensing and full-field imaging systems, and (iii) consideration of both the thin-lens (where the focal length of the CRL far exceeds its length) and thick-lens conditions (where this approximation is no longer valid).

We present a formalism for CRL-based imaging systems utilizing an RTM approach to model archetypal X-ray imaging systems in a lens-by-lens manner, thus accounting for both thin- and thick-lens conditions. Attenuation by the lens material is calculated using RTMs to trace the ray position through the CRL. We provide exact analytical expressions for focal length, numerical aperture, spatial resolution, vignetting and chromatic aberration among other key optical parameters. These expressions form the basis of an efficient parametric optimization of the CRL and imaging geometry, which ultimately provides suggestions for future lens development routes.

## 2. RTM formalism for CRLs   

### 2.1. Assumed CRL and lenslet geometry   

**Figure 1:**
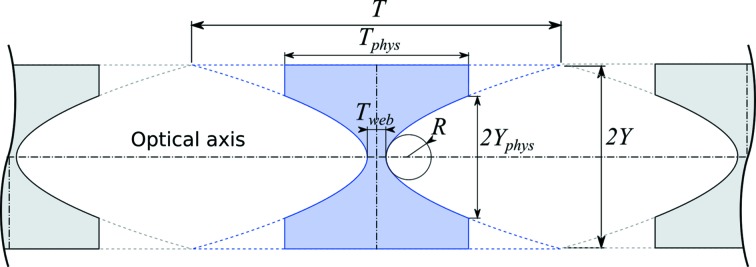
CRL and lenslet geometry assumed in this formalism. A single refracting lenslet element is shown in blue, annotated with symbolic dimensions.

This formalism assumes a one-dimensional (1D) focusing geometry valid for both axisymmetric two-dimensional and planar 1D CRLs. The CRL is comprised of *N* identical parabolic and non-kinofirm lenslets (Fig. 1 ▸), each with radius of curvature *R*, aperture 2*Y* and center-to-center distance between successive lenslets *T* such that 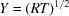. For manufacturing reasons, lenslets have a small distance between the parabolic apices (*i.e.* a web thickness) 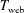 that affects attenuation. There may also be a gap between adjacent lenslets, implying that the physical lenslet thickness 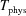 is less than *T*. Such geometries limit *Y* and necessitate defining the physical aperture 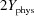 such that 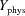 = 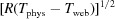.

### 2.2. Background to the RTM approach and focusing behavior   

RTM analysis is a paraxial ray-tracing approach that assumes all rays propagate nearly parallel to the optical axis. It does not intrinsically consider diffraction and total reflection; however, these may be introduced *ad hoc*. The approach treats each photon as a ray with transverse position *y* and angle to the longitudinal optical axis α, within an optical system defined by a matrix 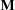 (*i.e.* an RTM) that transforms an incident ray 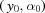 into an exit ray 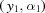,
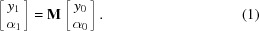
RTMs of compound systems may then be determined by multiplying the RTMs for the individual optical components. Previous RTM analyses of CRLs (Protopopov & Valiev, 1998 ▸; Pantell *et al.*, 2003 ▸; Poulsen & Poulsen, 2014 ▸) show that a single refractive X-ray lenslet can be described by three such components: a free-space propagation by *T*/2, a refracting thin lens with focal length *f* = 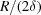 (where δ is the refractive decrement) and a final free-space propagation by *T*/2. Because *f* is many times larger than *T*, each lenslet behaves like an ideal thin lens with the following transfer matrix,
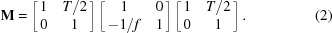
As the CRL is a linear array (*i.e.* a stack) of identical lenslets, its transfer matrix is 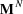 = 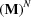. This can be calculated through the matrix eigendecomposition theorem (Poulsen & Poulsen, 2014 ▸) (see §S1 of the supporting information for derivation),
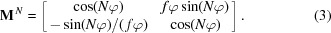
Within this paraxial treatment, the parameter φ can be expressed as 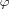 = 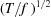 = 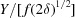. Thus, 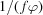 is the refractive power of the CRL per unit length (Lengeler *et al.*, 1999 ▸) while 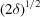 is the critical angle for total external reflection (Schroer & Lengeler, 2005 ▸).

The trigonometric terms in equation (3) imply periodicity with respect to 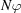. While attenuation by the lens means that CRLs practically operate within the first half-period (*i.e.* 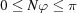), optical behavior differs markedly between the thin-lens limit (*i.e.* 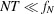 and correspondingly 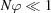) and the general thick lens case (*i.e.* all values of 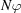). This formalism provides both cases in order to give straightforward access to the most common and important optical parameters for the vast majority of CRL geometries.

From equation (3), the focal length of the CRL as measured from its exit surface is given by the following two expressions (derived in §S2 of the supporting information), which are identical to those given in the literature (Poulsen & Poulsen, 2014 ▸; Lengeler *et al.*, 1999 ▸),
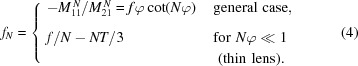



### 2.3. Ray transfer path   

In order to predict the attenuation of the rays as they traverse the CRL, the RTM approach must be extended. Specifically, we require an expression for the position and angle of a given ray at the *center* of the *n*th lenslet 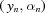 as a function of its incident state 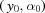. To this end, we compute the RTM of the CRL *after* the *n*th lenslet and back-propagate by *T*/2,
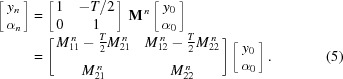
Inserting from equation (3) and simplifying (see §S3 of the supporting information), 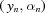 are then 
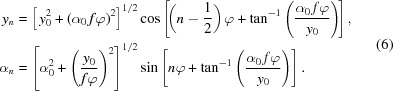
Within the CRL, all rays have a sinusoidal trajectory that varies with distance *nT* with a period of 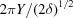. The physical aperture of the lenslets 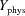 bounds this trajectory however, imposing the following criteria for participation in the focusing process,
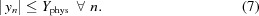
Rays may be excluded due to total reflection by the parabolic lenslet surfaces. In this case, the criteria for participation is 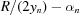 > 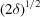. However, we note that, at typical X-ray energies, such reflection effects are only significant for lenslet geometries with impractically large values of 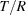. As such, we do not consider them further.

### 2.4. Attenuation in CRLs   

**Figure 2:**
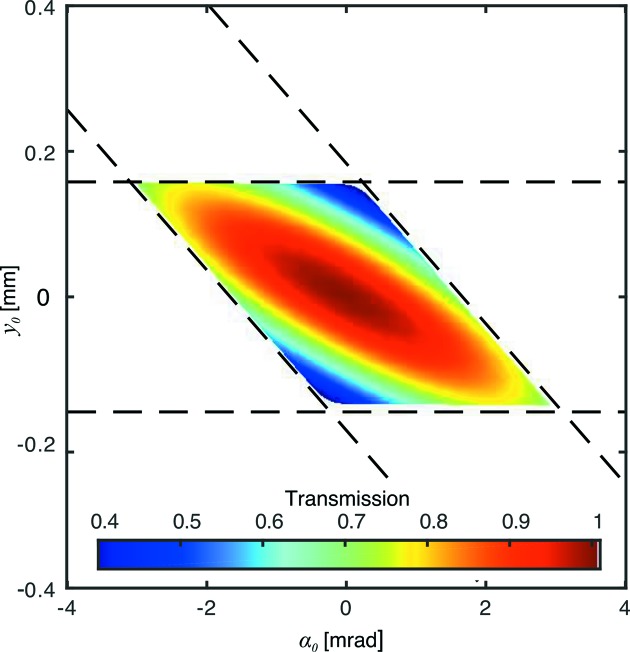
Transmission function 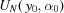 for a typical beryllium CRL at 17 keV with parameters 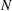 = 50, 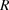 = 50 µm, 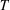 = 2 mm, 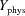 = 0.5 mm. The white region represents 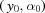 values excluded due to the constraints in equation (7), which can be approximated by two pairs of dashed lines corresponding to the entrance (horizontal pair) and exit (slanted pair) of the CRL.

The attenuation *U* of a ray passing through a single lenslet at a distance *y* from the optical axis depends on the absorption coefficient μ of the lens material and the local material thickness 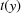 = 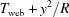. Since the paraxial approximation implies that the variation of *y* and 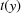 within the lenslet is negligible, the attenuation of the X-rays by the absorbing lenslet can be simply expressed using the Beer–Lambert law,
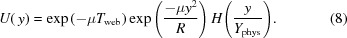
Here, *H* is a box function of width 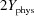 that enforces the criteria in equation (7). The cumulative transmission 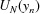 of a ray as it travels through *N* lenslets is then the product of the individual attenuation contributions from each lenslet,
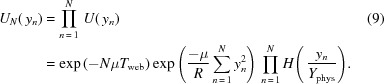
The central expression 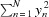 is a geometric sum that can be solved analytically (see §S4 of the supporting information). As all 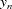 are a linear function of 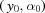, 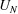 has a Gaussian dependence on both parameters. Combined with the conditions imposed by *H*, this results in a bounded two-dimensional Gaussian transmission distribution, as shown in Fig. 2 ▸.

### 2.5. Effective aperture and transmission efficiency of a CRL   

The spatial acceptance function for a homogeneous and parallel incident beam can then be calculated from 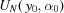 by inserting 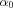 = 0 into equation (6) (see §S5 of the supporting information). This results in a 1D Gaussian transmission profile in 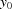 with a root mean square (RMS) value 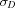 of
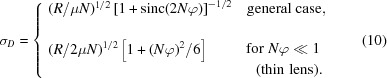
From this, we can calculate the effective aperture 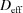: the diameter of a circular pinhole with the same total transmitted intensity as a (two-dimensional) CRL made from rotationally symmetric paraboloids. For all values of 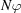, we find
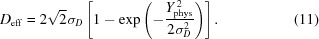
The transmission efficiency is then given by
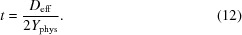
These expressions for 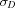, 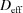 and *t* provide a convenient means to compare the attenuation-limited properties of CRLs independent of the optical system they are operating in. Coupled with the expression for 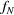 [equation (4)], they constitute the simplest way to characterize CRL performance.

## 3. CRL-based imaging systems   

### 3.1. The imaging condition   

**Figure 3:**
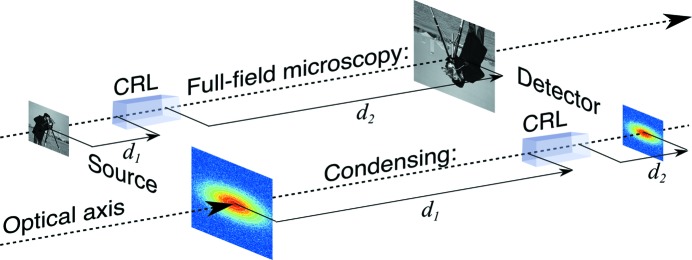
Comparison of CRL-based imaging systems. Full-field transmission X-ray microscopy (rear) *versus* a condensing system that demagnifies a (typically Gaussian) X-ray source (front).

The general imaging case describes both condensing (Schroer *et al.*, 2005 ▸) and full-field (Lengeler *et al.*, 1999 ▸) geometries comprising a source, a lens (either as an objective or condenser) and an image/detection plane. Note that these geometries are mathematically identical; condensing can be seen as full-field imaging of the source with a magnification ratio of less than one (shown schematically in Fig. 3 ▸).

In an imaging configuration, a ray originating from the source plane at 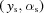 travels a distance 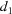 to the objective, where it is transformed by the CRL RTM 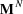 before travelling a distance 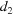 to a point 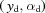 on the detector plane. This transformation between 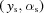 and 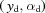 can be expressed as
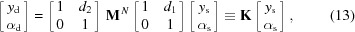
where 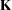 is the matrix
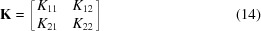
whose components can be expressed in terms of 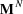 as
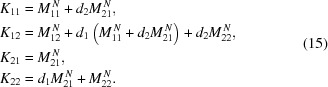
The imaging condition implies that 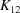 = 0, which leads to the general imaging formula (derivation in §S6 of the supporting information)
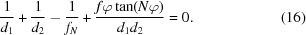
The magnification of the imaging system 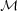 (a positive number) is defined by 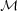 = 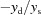 = 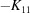. Combined with equation (16), this gives a set of two equations, which can be solved to give exact expressions for magnification 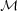 and the imaging distances 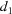 and 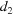,
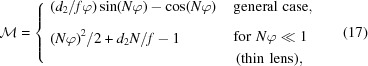


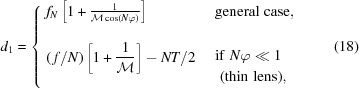


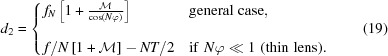
Note that 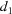 and 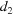 can never be negative in equations (18) and (19), resulting in the following conditions on 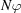,
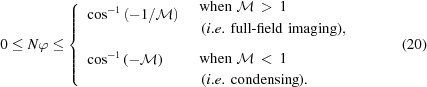
The number of lenses *N* necessary to achieve a given magnification 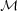 and source-to-detector distance *L* = 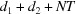 can be calculated by rewriting the imaging formula [equation (16)] as follows,
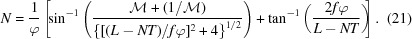
Despite a factor in the denominator, *N* can nonetheless be evaluated iteratively since 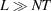 (solution by fixed-point method). Furthermore, as *N* must be an integer number, 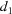 and 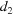 must be adjusted to fulfill the imaging condition. Consequently, a small deviation of the magnification 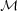 from the target value must be accepted.

We observe that equation (13) can also be used to describe the geometry of the back focal plane, which for these imaging systems is located at 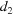 = 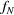. The intensity distribution at the back focal plane is closely related to the Fourier transform of the object in the sample plane, and as such can be used as a means for quantifying micro- and nano-scale periodicity (Ershov *et al.*, 2013 ▸). Inserting 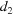 = 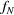 into equations (13)–(15) gives 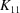 = 0 and
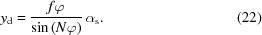
Hence, X-rays emerging from the sample plane at the angle α_s_ will converge to position 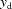 in the back focal plane.

### 3.2. Attenuation in imaging systems   

The spatial and angular acceptance of the CRL are defining characteristics of imaging systems, jointly defined by the attenuation properties of the lens and the specific geometry of the system. To find expressions for them, we first use equation (6) to provide 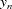 as a function of a ray’s position and angle in the source plane, 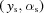,
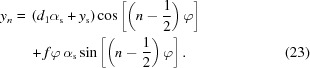
Inserting this into 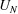 [equation (9)] gives the complete acceptance function of the imaging system, which can be rewritten as follows (derivation in §S7 of the supporting information),
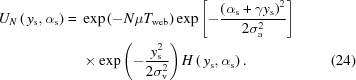
Notably, this is the product of a prefactor, a Gaussian with RMS 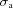 and offset coefficient γ describing the system’s angular acceptance, another Gaussian with RMS 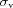 decribing the system’s spatial acceptance (*i.e.* vignetting) and a box function 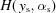 representing the system’s physical aperture. This physical aperture 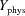 imposes a sharp cut-off to the acceptances [see equation (7)]. In the same manner as equation (9), this is represented by 
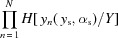
 which, from equation (23), is well approximated by
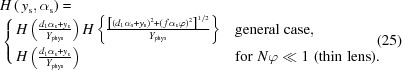
The angular acceptance, defined by its RMS 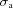 and offset 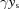, describes the range of angles over which the lens collects radiation emitted from a point 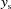 on the source plane and ultimately defines the theoretical image resolution achievable by the system. At any point in the field of view (*i.e.* at any 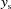), the system will have a Gaussian angular acceptance with RMS 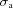 given by
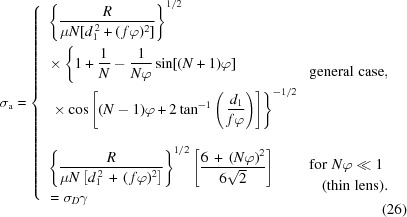
The offset coefficient γ describes the median angle accepted by the lens at a particular point on the sample plane 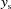. Notably, in the thin-lens case the characteristic distance 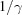 is the weighted average of the imaging distance 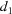 and the period of the sinusoidal divided by 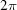. The full derivation for γ is provided in §S7 of the supporting information.

The spatial acceptance has RMS 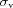 and describes the reduction in brightness from the center of the optical axis towards its periphery, *i.e.* the maximum achievable field-of-view of the system. This is defined as the total acceptance of the system integrated across all incident angles 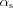. The general expression for the RMS of the vignetting function is given by
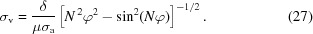
The leading term in the thin-lens limit is of the order 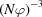, meaning that the vignetting does not originate from the material attenuation at the thin-lens limit. Instead, the thin-lens vignetting function, 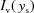, is defined by the physical aperture of the small stack of lenses,
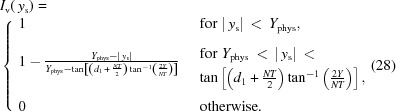
The numerical aperture (NA) is a dimensionless number characterizing the range of angles accepted by the imaging system, and is therefore naturally related to 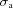. The parameter is regularly used in the context of visible-light systems where this range is sharply defined by the physical aperture of the system. However, the Gaussian nature of the X-ray acceptance function of typical thick CRLs makes such approaches inappropriate. Instead, the definition used for Gaussian laser systems is used here (Born & Wolf, 1999 ▸), in which the NA at a given position 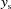 is given in terms of the ray angle where the transmission drops to 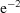,
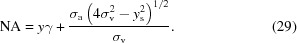
Note that this reduces to NA = 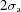 at the center of the field-of-view.

### 3.3. Spatial resolution   

**Figure 4:**
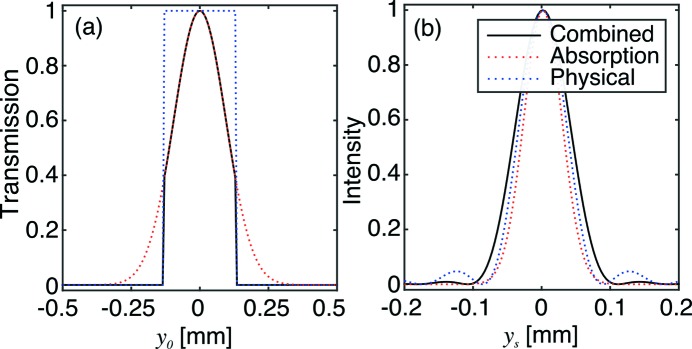
Transmission function (*a*) and PSF (*b*) for a typical beryllium CRL at 17 keV with parameters 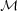 = 20, 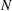 = 50, 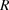 = 50 µm, 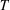 = 2 mm, 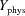 = 0.15 mm. The solid black line corresponds to the cumulative response of the CRL, while the dotted red and blue lines correspond to the contributions from the material absorption and physical aperture, respectively.

Analytical expressions for the spatial resolution of the magnified image can now be derived from the magnification 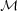 and the angular acceptance 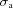 of the imaging system. Spatial resolution may be defined as the minimum distinguishable distance 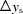 between two points at the source/sample plane (Born & Wolf, 1999 ▸). The degree of blurring of these points due to diffraction and the aberration inherent in lens-based optical systems is described by the point spread function (PSF), which can be calculated from the Fourier transform of the CRL pupil function (Born & Wolf, 1999 ▸). Neglecting aberration, the effective pupil function 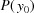 for the center of the image can be derived from equation (24) by substituting 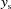 = 0 and 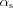 = 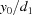, where 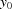 is the ray position at the CRL entrance (*i.e.* 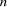 = 0) (see §S8 of the supporting information),
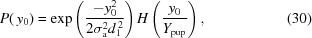
where 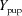 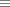 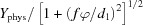. For a wavenumber *k* = 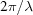 at X-ray wavelength λ, and ignoring constant prefactors, the point-spread intensity function at the source plane, 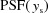, is then given by
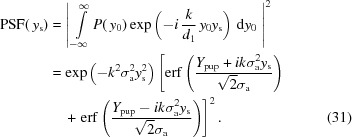
The PSF has two components: a Gaussian with RMS σ_PSF_ = 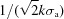 and a complex term comprising two error functions representing the effect of the physical aperture 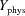. The relative contributions of these components are shown in Fig. 4 ▸. Notably, in the absence of a physical aperture (*i.e.* if the CRL is limited by the attenuation of the lens material), only the Gaussian component of the PSF remains.

In classical optical systems, the resolution is often defined by the Rayleigh criterion, where two PSFs are regarded distinguishable when the maximum of one PSF coincides with the first minimum of the other (Born & Wolf, 1999 ▸). However, this is inappropriate in the case of a Gaussian or near-Gaussian PSF such as for CRLs, where such a minimum may not be present. Instead, we propose that the resolution be defined by the separation distance 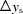 between two PSFs corresponding to a contrast ratio of *C* (where *C* is small when the contrast is poor). Using equation (32), this can be determined by numerically solving
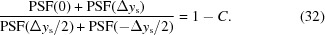
In the case of absorption-limited (*i.e.* Gaussian) CRLs, this gives a function in terms of λ, 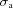 and *C*,
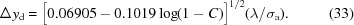
The value of *C* necessary to distinguish details depends on the sampling statistics. In the case of low-intensity (*e.g.* dynamic) measurements, *C* should be greater than the equivalent for the Rayleigh criteria (approximately 0.26).

### 3.4. Chromatic aberration   

**Figure 5:**
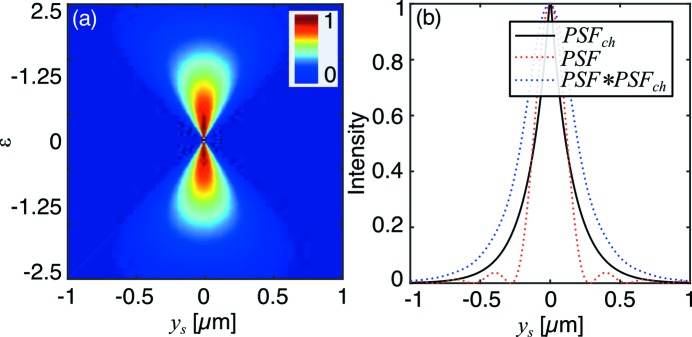
Spatio-chromatic intensity distribution for a typical beryllium CRL at 17 keV with parameters 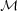 = 20, 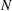 = 50, 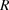 = 50 µm, 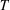 = 2 mm, 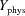 = 0.5 mm at 17 keV (*a*). Point-spread function of the same Be CRL and configuration, assuming a bandwidth of 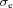 = 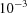 (*b*).

Imaging with a wide energy bandwidth can be advantageous due to the significant increase in photon flux. The bandwidth is ultimately defined by the type of X-ray source and conditioning optics: the raw spectrum from an undulator (*i.e.* the pink beam) is typically of the order of 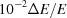, while monochromators can provide bandwidths from 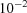 to 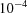. As most conditioning schemes use a diffraction-based monochromator, the energy spectrum is typically Gaussian. As such, the spectrum can be defined around a nominal energy 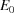 and intensity 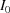, in terms of the energy perturbation ∊, defined by 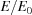 = 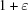 and the RMS bandwidth 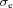,
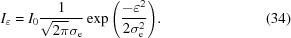
Small energy perturbations alter δ from its nominal value 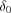 according to 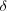 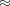 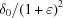. Since the focal length of the lenslets depend on δ, CRLs are chromatic by nature. Under an ideal imaging condition at 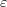 = 0, a ray departing from the center of the sample plane will strike the center of the detector plane, *i.e.* 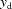 = 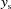 = 0. At a slightly different photon energy, 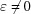; however, the same ray will be displaced in the detector plane, 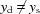 = 0. The position, 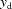, at which an incident ray from 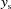 = 0 strikes the detector, can be approximated by inserting the chromatic expression for δ above into equation (13), and Taylor expanding to first order in ∊,
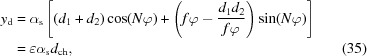
where 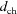 is a distance term for the imaging system given in terms of the nominal values for φ and *f* by
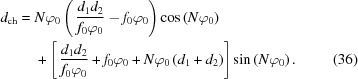
The ray is attenuated depending on its incident angle 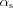. Noting from equation (35) that this angle may be written as 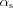 = 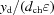, the spatio-chromatic intensity distribution of the rays on the detector plane, 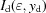, is then determined from equations (31) and (34) as (see §S9 of the supporting information)
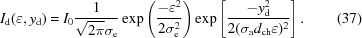
This distribution (Fig. 5 ▸) is consistent with experimental results in the literature (Falch *et al.*, 2016 ▸) and illustrates that the chromatic spread of intensity becomes broader as ∊ deviates further from zero.

The point-spread function as a result of this chromatic behavior, 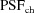, is determined by integrating 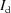 across ∊, and normalizing 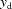 by the nominal magnification 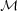,
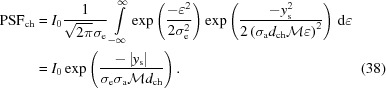
Notably, this is a Laplace distribution described by the characteristic width 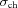,
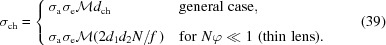
The combined PSF from a chromatic and diffraction-limited system will be a convolution of the two PSFs in equations (31) and (38), shown in Fig. 5 ▸. While this does not have a convenient analytical expression, it can be readily solved by numerical methods.

## 4. Optimization and the thick-lens limit   

The analytical expressions for key optical parameters such as spatial resolution, vignetting and aberration provide a foundation for numerical optimization. In the most general case, the aim is to optimize some figure-of-merit function with respect to X-ray wavelenth (λ), CRL geometry (*R, T, N*) and imaging geometry (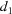, 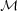 or *L*, 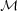) for a given material (δ, μ).

**Figure 6:**
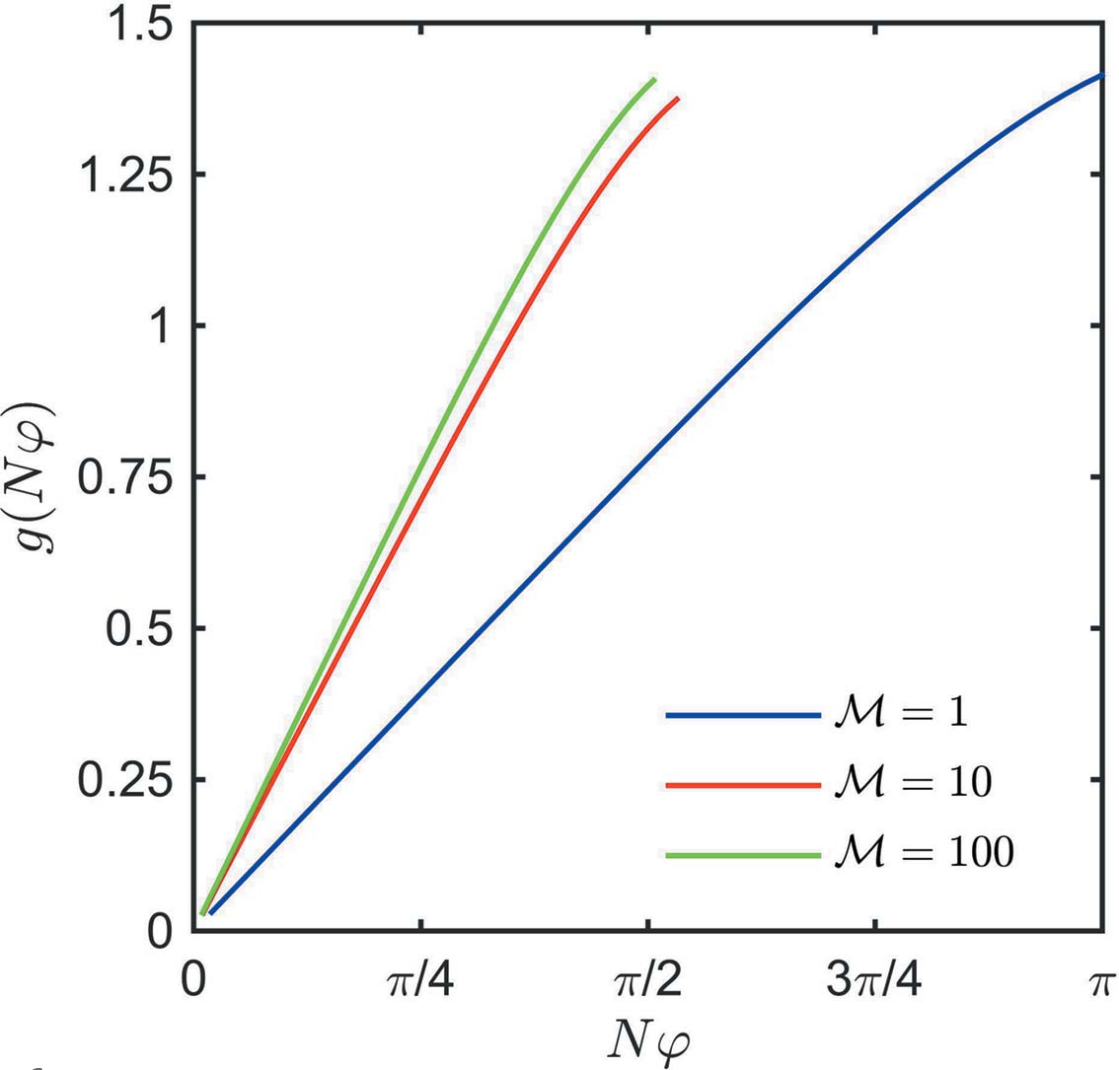
Value of 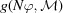 for different values of 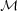.

In imaging systems, optimizing NA and 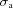 can improve the diffraction- and chromatic-limited resolution [equations (31) and (38)] while increasing transmission efficiency. Because 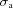 is directly coupled to 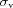 [equation (27)], it also determines the image vignetting profile. The multidimensional problem of optimizing 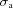 for a given wavelength can, to a good approximation, be greatly simplified by assuming that *N* is large, *i.e.* 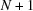 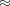 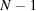 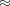 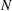. Then equation (26) becomes
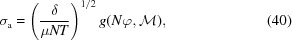
where 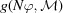 is the expression 
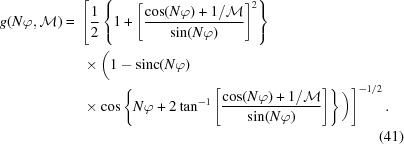
Equation (40) confirms the well known belief that optical performance (*i.e.* 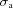) is maximized when 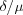 is large (Snigirev *et al.*, 1996 ▸). However, the expression also dictates that 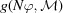 be maximized too. Plotting 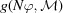 for the allowable range of 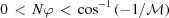 and some typical 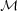 values (Fig. 6 ▸) shows that *g* reaches a maximum value at large 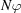.

Correspondingly, there is a global optimum 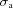 at 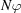 = 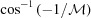. For typical values of 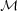, this thick-lens limit is approximately at 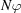 = 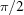, and is associated with some unusual imaging geometries,
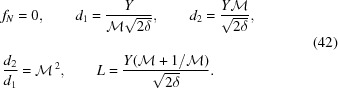
As discussed, the angular acceptance 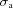 reaches its maximum at the thick-lens limit close to 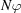 = 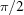. As the magnification approaches infinity, 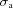, 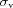 and 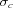 are
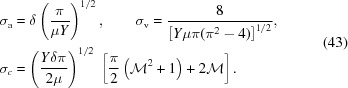
The first of these expressions show that the lenslet aperture *Y* is the critical geometrical term that fundamentally limits the optimal angular acceptance of CRL-based imaging systems. Similarly, *Y* is also a fundamental term for the vignetting profile and the chromatic spread. These expressions clearly demonstrate the need to miniaturize the lenslet geometry, as reducing *Y* is the only path to increasing the acceptance for a given set of 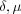 parameters. Most importantly, the fact that *Y* = 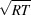 implies that there are a range of lenslet geometries that will satisfy the following conditions for optimization,
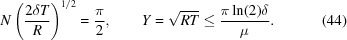



## 5. Discussion   

Following previous RTM formalisms (Protopopov & Valiev, 1998 ▸; Pantell *et al.*, 2003 ▸; Poulsen & Poulsen, 2014 ▸), this implementation is shown to be a versatile and effective tool for many aspects of the optimization of CRL-based X-ray imaging systems. It is particularly suitable for calculating optical parameters for systems operating in the thick-lens condition. Being a linear formalism, the approach does not account for spherical or higher-order aberrations; however, some effects of diffraction and refraction can be included. Similarly, CRL geometries where the lenslet profile varies along the CRL thickness direction [*i.e.* adiabatic lenses (Schroer & Lengeler, 2005 ▸; Chen *et al.*, 2014 ▸)] are not intrinsically accounted for, but can be calculated numerically by substituting a function of, for example, 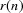 or 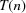 for *R* or *T*, respectively. In the case of astigmatic lenses such as two-dimensional CRLs produced by interdigitating 1D chips (Simons *et al.*, 2016 ▸), the RTM formalism can be readily extended to either two 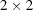 systems or a single 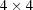 system that can calculate the astigmatism analytically.

The optimization demonstrated in this work was largely unconstrained and thus represents a simplified imaging configuration. Applying the practical constraints of real beamline hutch geometries therefore requires additional constraints to the optimization. However, a significant advantage of the RTM formalism is its versatility and mathematical simplicity compared with, for example, Monte Carlo and wave propagation methods. As such, the number of free parameters could be increased without necessarily resulting in impractical computation times.

**Figure 7:**
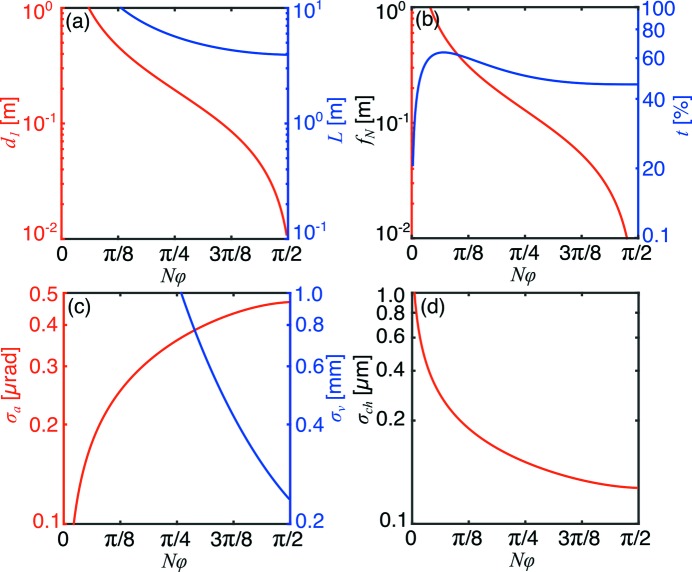
Critical optical parameters as a function of 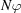 for a typical imaging system with 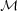 = 20, 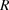 = 50 µm, 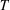 = 2 mm, 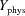 = 0.5 mm at 17 keV with 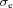 = 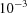. Shown are the sample–objective and total imaging distances (*a*), the CRL focal length and transmission efficiency (*b*), the angular acceptance and vignetting (*c*) and chromatic aberration width (*d*).

A major result of this work is that the spatial resolution is globally optimized at the thick-lens limit: 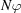 = 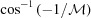 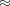 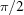. This can be observed directly in Fig. 7 ▸, where 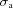 is maximized and 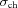 is minimized at this limit. Furthermore, the field-of-view and imaging geometries also remain practical at this thick-lens limit: 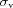 is above 200 µm, *L* is 4 m and 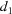 is 10 mm at the optimum 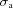 in the example presented in Fig. 7 ▸.

The majority of CRL microscopes described in the literature (Simons *et al.*, 2015 ▸) operate quite far from this optimimum configuration, implying that there may be significant resolution gains from increasing the focal power of CRLs beyond their current state. This is particularly true in the case of pink-beam X-ray imaging, where the chromatic blurring is minimized at the thick-lens limit.

The formalism indicates that smaller *R* and *T* will offer superior performance, making a strong case for miniaturizing the lenslet geometry. However, the optimization also shows that the global maximum for 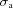 can be reached by *any* lens geometry that satisfies equation (44). Thus, significant gains can still be had by reducing *R* or *T* alone. This is particularly important when manufacturing CRLs, as certain processes may prevent the realisation of specific lenslet geometries. For example, the performance of a CRL comprising indented two-dimensional metal lenslets may be improved by reducing the thickness of the lenslet rather than reducing the parabola radius, thus avoiding the shape-error and aberration that usually accompanies the production of small radii.

This formalism and optimization approach are key for the design of X-ray imaging experiments and instruments. The global optimum at small imaging distances implies that imaging systems with a small footprint will be optically superior. Such systems can offer improved mechanical stability and ultimately higher spatial resolution, and are therefore recommended on the basis of this work. The calculations performed here also shed light on the expected gains from upcoming fourth-generation synchrotron sources, which are characterized by unprecedented brilliance and a smaller energy bandwidth. As demonstrated in Fig. 7 ▸, the contribution of chromatic aberration to the PSF is expected to significantly decrease for lenses whose geometry approaches the thick-lens limit. We therefore predict that high-speed three-dimensional X-ray microscopy may be possible without the use of a monochromator.

## 6. Conclusions   

This work describes a means of calculating and optimizing the optical properties of CRLs and some of the most common CRL-based imaging systems. Specifically:

(i) We developed a formalism with closed analytical expressions for key optical parameters, such as focal length, imaging distances, vignetting, spatial resolution and chromatic aberration. The expressions are relevant to the vast majority of X-ray microscopes, and pertain to both full-field imaging and condensing systems as well as thin- and thick-lens conditions.

(ii) We carried out an efficient global optimization on the archetypal X-ray imaging system. While this example was geometrically unconstrained, we note that practical limits to, for example, imaging distances can be easily incorporated.

(iii) The optimization identified that the optimum spatial resolution for any material and energy will occur at the thick-lens limit of 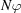 = 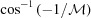. Hence, manufacturing CRLs at this thick-lens limit is an opportunity for resolution enhancement.

(iv) This implies that the optimum resolution may be reached with many different CRL geometries. This creates a significant opportunity for tailoring CRL geometries for specific manufacturing processes, *e.g.* lithography or indentation, *etc*.

(v) Chromatic aberration is reduced near this thick-lens limit. This means that larger energy bandwidths can be used with thick lenses, potentially increasing imaging flux and providing a new opportunity for high-speed dynamic imaging experiments.

Ultimately, we hope that the expressions and optimization approach described here can be applied to improve the performance and design of full-field X-ray microscopes. For existing lens materials and manufacturing technology such as the example given in Fig. 7 ▸, one can expect such optimizations to yield improvements in numerical aperture and spatial resolution by a factor of two or more, while simultaneously allowing greater X-ray energy bandwidth and flux. With the advent of many new instruments, both for the laboratory and in synchrotrons, we believe this capability is of significant contemporary relevance.

## Supplementary Material

Supporting derivations for key equations in the main text.. DOI: 10.1107/S160057751602049X/ie5162sup1.pdf
