# Supplementary material for: Simulating and optimizing compound refractive lens-based X-ray microscopes
Source: J Synchrotron Radiat. 2017 Feb 20;24(Pt 2):392–401. doi: 10.1107/S160057751602049X (PMC5330290; doi:10.1107/S160057751602049X)
Supplement: Supplementary file 1 [file s-24-00392-sup1.pdf]

JOURNAL OF
SYNCHROTRON
RADIATION

Volume 24 (2017)

Supporting information for article:

Simulating and optimizing compound refractive lens-based X-ray microscopes

Hugh Simons, Sonja Rosenlund Ahl, Henning Friis Poulsen and Carsten Detlefs

SUPPORTING INFORMATION: Simulating and optimizing compound refractive lens-based x-ray microscopes

Hugh Simons,[1] Sonja Rosenlund Ahl,[1] Henning Friis Poulsen,[1] and Carsten Detlefs[2]
[1]*Department of Physics, Technical University of Denmark, Lyngby 2800 kgs, Denmark*
[2]*European Synchrotron Radiation Facility, 71 Avenue des Martyrs, Grenoble 38000, France*
(Dated: June 25, 2016)

PACS numbers:

S1: Derivation of the CRL transfer matrix

The transfer matrix of the CRL, $\mathbf{M}^N$ is the combined behaviour of N such lenslets. This can be calculated through the matrix eigendecomposition theorem, where $\mathbf{P}$ is a matrix comprising the eigen*vectors* of $\mathbf{M}$ and $\mathbf{D}$ is a diagonal matrix comprising the eigen*values* of $\mathbf{M}$.

$$\mathbf{M}^N = \mathbf{MM}...\mathbf{M} = (\mathbf{M})^N = \mathbf{PD}^N\mathbf{P^{-1}} \tag{1}$$

The eigenvalues $E_\pm$ - which are a complex conjugate pair if $4f > T$ - may then be expressed as:

$$E_\pm = 1 - \frac{T}{2f} \pm i\sqrt{1 - \left(1 - \frac{T}{2f}\right)^2} = \cos(\varphi) \pm i\sin(\varphi) = \exp\left(\pm i\varphi\right) \tag{2}$$

where φ is the phase angle of the complex eigenvalues, which may be expressed as:

$$\varphi = \tan^{-1}\left(\frac{\sqrt{1-\left(1-\frac{T}{2f}\right)^2}}{1-\frac{T}{2f}}\right) \approx \sqrt{\frac{T}{f}} \tag{3}$$

The corresponding eigenvectors $\mathbf{r}_\pm = (y_\pm, \alpha_\pm)$ are given by $y_\pm = \mp i\alpha_\pm f\sin\varphi$. From Eq. 2 the expression for $\mathbf{M}^N$ then becomes

$$\begin{aligned}\mathbf{M^N} &= \begin{bmatrix} 1 & 1 \\ \frac{1}{if\sin\varphi} & \frac{-1}{if\sin\varphi}\end{bmatrix}\begin{bmatrix}\exp\left(-iN\varphi\right) & 0 \\ 0 & \exp\left(iN\varphi\right)\end{bmatrix}\begin{bmatrix} 1 & 1 \\ \frac{1}{if\sin\varphi} & \frac{-1}{if\sin\varphi}\end{bmatrix}^{-1} \\ &= \begin{bmatrix}\cos\left(N\varphi\right) & f\sin\varphi\sin\left(N\varphi\right) \\ \frac{-\sin\left(N\varphi\right)}{f\sin\varphi} & \cos\left(N\varphi\right)\end{bmatrix}\end{aligned} \tag{4}$$

Note also that the basic approximation of the RTM approach implies that

$$\begin{aligned}\sin\varphi = \varphi = \sqrt{\frac{T}{f}} &= \frac{1}{\sqrt{2\delta}}\frac{Y}{f} \\ f\sin\varphi = f\varphi = \sqrt{Tf} &= \frac{Y}{\sqrt{2\delta}}\end{aligned} \tag{5}$$

Here, $\sqrt{2\delta}$ is the critical angle for total external reflection and the numerical aperture for an ideal elliptical lens[1] corresponding to $R = T$ in the parabolic approximation. If there is no space between lenslets, Y/f is half the numerical aperture of one lenslet and $1/(f\varphi)$ is the refractive power of the CRL per unit length[2].

Hence, from Eqs. 4and 5, $\mathbf{M}^N$ can then be expressed as:

$$\mathbf{M}^N = \begin{bmatrix}\cos\left(N\varphi\right) & f\varphi\sin\left(N\varphi\right) \\ \frac{-\sin\left(N\varphi\right)}{f\varphi} & \cos\left(N\varphi\right)\end{bmatrix} \tag{6}$$

S2: Derivation of the expressions for focal length

The focal length is the distance from the lens exit f_N at which a refracted ray with initial angle $\alpha_0 = 0$ will intersect with the optical axis at $y = 0$. This can be conveniently extracted from $\mathbf{M}^N$[3]:

$$f_N = -\frac{M_{11}^N}{M_{21}^N} = f\varphi \cot(N\varphi) \tag{7}$$

Notably, f_N is measured from the exit point of the CRL along the optical axis, $NT/2$ from the CRL centre. For small $N\varphi$ the expression above approximates to $f_N = \frac{R}{2\delta N} - \frac{NT}{3}$, for which negligible total lens thickness NT reproduces equivalent expressions derived by alternative means, e.g. from[2].

S3: Derivation of the ray path within CRLs

To calculate the position of a ray at the centre of the nth lenslet, we determine the RTM of the CRL after the lenslet and then back-propagate by half a lenslet distance (i.e. $-T/2$). The ray vector (y_n, α_n) as a function of the incident ray (y_0, α_0) is then:

$$\begin{bmatrix} y_n \\ \alpha_n \end{bmatrix} = \begin{bmatrix} 1 & -T/2 \\ 0 & 1 \end{bmatrix} \mathbf{M}^n \begin{bmatrix} y_0 \\ \alpha_0 \end{bmatrix} = \begin{bmatrix} M_{11}^n - \frac{T}{2}M_{21}^n & M_{12}^n - \frac{T}{2}M_{22}^n \\ M_{21}^n & M_{22}^n \end{bmatrix} \begin{bmatrix} y_0 \\ \alpha_0 \end{bmatrix} \tag{8}$$

Meaning that the lateral position y_n is given by:

$$\begin{aligned} y_n &= y_0 \left(M_{11}^n - \frac{T}{2} M_{21}^n \right) + \alpha_0 \left(M_{12}^n - \frac{T}{2} M_{22}^n \right) \\ &= y_0 \left(\cos(n\varphi) + \frac{T \sin(n\varphi)}{2f\varphi} \right) + \alpha_0 \left(f\varphi \sin(n\varphi) - \frac{T}{2} \cos(n\varphi) \right) \\ &= y_0 \cos\left(\left(n - \frac{1}{2} \right) \varphi \right) + \alpha_0 f\varphi \sin\left(\left(n - \frac{1}{2} \right) \varphi \right) \end{aligned} \tag{9}$$

Since:

$$\begin{aligned} \cos(n\varphi) + \frac{T\sin(n\varphi)}{2f\varphi} &= \cos(n\varphi) + \frac{\varphi}{2}\sin(n\varphi) = \cos\left(\left(n - \frac{1}{2}\right)\varphi\right) \\ f\varphi\sin(n\varphi) - \frac{T}{2}\cos(n\varphi) &= f\varphi\left(\sin(n\varphi) - \frac{\varphi}{2}\cos(n\varphi)\right) = f\varphi\sin\left(\left(n - \frac{1}{2}\right)\varphi\right) \end{aligned} \tag{10}$$

Trigonometric identities (see Appendix A) then give:

$$y_n = \sqrt{y_0^2 + (\alpha_0 f\varphi)^2} \sin\left(\left(n - \frac{1}{2}\right)\varphi + \tan^{-1}\left(\frac{y_0}{\alpha_0 f\varphi}\right)\right) \tag{11}$$

All rays through the CRL will therefore have a sinusoidal trajectory with a period of $\frac{2\pi Y}{\sqrt{2\delta}}$ that is proportional to the aperture Y. Likewise, for α_n:

$$\alpha_n = \frac{1}{f\varphi}\sqrt{y_0^2 + (\alpha_0 f\varphi)^2} \sin\left(n\varphi - \tan^{-1}\left(\frac{y_0}{\alpha_0 f\varphi}\right)\right) \tag{12}$$

If at any point at the trajectory, $| y_n |> Y_{\text{phys}}$, the ray will not be refracted, but either absorbed or contribute to the general background after the lens. From this, the criterion for any ray to participate in the focusing/imaging process becomes:

$$| y |_{max} \leq Y_{\text{phys}} \tag{13}$$

The imaging condition implies that there at most can be one extremum in practical systems. Hence, the maximum value of y, y_{max} is:

$$| y |_{max} = \max\left(| y_0 |, | y_N |, \sqrt{y_0^2 + (\alpha_0 f\varphi)^2} \right) \tag{14}$$

S4: Derivation of the attenuation profile

For a single lenslet, the attenuation U of a ray passing through a lenslet with linear absorption coefficient μ and local thickness t at perpendicular distance y from the optical axis can be described by the Beer-Lambert law and a box function H, which fully attenuates any ray that exceeds the physical aperture Y_{phys}:

$$U(y) = \exp\left[-\mu t(y)\right] \times H\left(\frac{y}{Y_{\text{phys}}}\right), \tag{15}$$

where the box function H is:

$$H(x) = \begin{cases} 1 & \text{for} |x| \leq 1 \\ 0 & \text{for} |x| > 1 \end{cases} \tag{16}$$

The paraxial assumption is that the rays propagate each lenslet at a constant distance to the optical axis. The parabolic profile of the lenslets then gives the thickness function $t(y)$:

$$t(y) = T_{\text{web}} + \frac{y^2}{R} \qquad \text{for} \qquad |\, y \,| < Y_{\text{phys}} \tag{17}$$

Thus, neglecting the effects of air between lenslets (i.e. assuming high energy x-rays), a single lenslet will attenuate the ray according to:

$$U(y) = \exp\left(-\mu T_{\text{web}}\right) \times \exp\left(\frac{-\mu y^2}{R}\right) \times H\left(\frac{y}{Y_{\text{phys}}}\right) \tag{18}$$

The cumulative attenuation $U_N(y)$ of a ray as it travels through N lenslets is then the product of the individual attenuation contributions from each lenslet:

$$U_N = \prod_{n=1}^{N} U(y_n) = \exp\left(-N\mu T_{\text{web}}\right) \times \exp\left(\frac{-\mu}{R}\sum_{n=1}^{N} y_n^2\right) \times \prod_{n=1}^{N} H\left(\frac{y_n}{Y_{\text{phys}}}\right), \tag{19}$$

where y_n is the position of the ray at the centre of the nth lenslet. The central expression for $\frac{\mu}{R}\sum_{n=1}^{N} y_n^2$ is a quadratic term, which can be expressed as:

$$\frac{\mu}{R}\sum_{n=1}^{N} y_n^2 = A_N \alpha_0^2 + B_N \alpha_0 y_0 + C_N y_0^2 \tag{20}$$

The coefficients A_N, B_N and C_N are geometric sums which, since $1 + \varphi^2 \approx 1$, have solutions to second order in φ (see Appendix for identities):

$$\begin{aligned} A_N &= \frac{\mu}{R}\sum_{n=1}^{N}\left(M_{12}^n - \frac{T}{2}M_{22}^n\right)^2 \\ &= \frac{N\mu(f\varphi)^2}{2R}\left(1 - \operatorname{sinc}(2N\varphi)\right) \\ &= \frac{N\mu T}{4\delta}\left(1 - \operatorname{sinc}(2N\varphi)\right) \end{aligned} \tag{21}$$

$$\begin{aligned} B_N &= \frac{\mu}{R}\sum_{n=1}^{N}\left(M_{11}^n - \frac{T}{2}M_{21}^n\right)\left(M_{12}^n - \frac{T}{2}M_{22}^n\right) \\ &= \frac{\mu}{2\delta}\left(1 - \cos(2N\varphi)\right) \end{aligned} \tag{22}$$

$$\begin{aligned} C_N &= \frac{\mu}{R}\sum_{n=1}^{N}\left(M_{11}^n - \frac{T}{2}M_{21}^n\right)^2 \\ &= \frac{N\mu}{2R}\left(1 + \operatorname{sinc}(2N\varphi)\right) \end{aligned} \tag{23}$$

Hence, the attenuation function can be expressed as a 2D tilted Gaussian function with clipped' boundaries defined by y_n/Y_{phys}:

$$U_N = \exp\left(-N\mu T_{\text{web}}\right) \times \exp\left(A_N\alpha_0^2 + B_N\alpha_0 y_0 + C_N y_0^2\right) \times \prod_{n=1}^{N} H\left(\frac{y_n}{Y_{\text{phys}}}\right), \tag{24}$$

S5: Derivation of physical and effective apertures

The physical aperture of the CRL corresponds to the spatial acceptance (i.e. in y) for a parallel incident beam (i.e. $\alpha_0 = 0$). When Y_{phys} is sufficiently large, the spatial acceptance has a Gaussian profile in y:

$$U_{\text{phys}} = \exp\left(-\mu N T\right) \exp\left(\frac{-y_0^2}{2\sigma_D^2}\right) \tag{25}$$

In such (common) cases, the physical aperture is given by the RMS σ_D:

$$\sigma_D = \frac{1}{\sqrt{2C_N}} = \sqrt{\frac{R}{\mu N}}\frac{1}{\sqrt{1+\text{sinc}(2N\varphi)}} \tag{26}$$

The effective aperture is the diameter D_{eff} of a cylindrical pupil function that has the same total transmission as the CRL[2]. Its expression is found by solving:

$$\pi\left(\frac{D_{\text{eff}}}{2}\right)^2 = 2\pi \exp\left(-\mu N T\right) \int_0^{Y_{\text{phys}}} \exp\left(\frac{-y_0^2}{2\sigma_D^2}\right) y_0 dy_0 \tag{27}$$

Hence:

$$D_{\text{eff}} = 2\sqrt{2}\sigma_D \exp\left(\frac{-\mu N T}{2}\right)\left[1 - \exp\left(\frac{-Y_{\text{phys}}^2}{2\sigma_D^2}\right)\right]^{\frac{1}{2}} \tag{28}$$

S6: Derivation of imaging geometries

In an imaging configuration, a ray originating from the sample plane at (y_s, α_s) travels through the objective to the detector plane at (y_d, α_d). This transformation between the two planes can be expressed as:

$$\begin{bmatrix} y_d \\ \alpha_d \end{bmatrix} = \begin{bmatrix} 1 & d_2 \\ 0 & 1 \end{bmatrix} \mathbf{M^N} \begin{bmatrix} 1 & d_1 \\ 0 & 1 \end{bmatrix} \begin{bmatrix} y_s \\ \alpha_s \end{bmatrix} \equiv \mathbf{K} \begin{bmatrix} y_s \\ \alpha_s \end{bmatrix} \tag{29}$$

Here, d_1 is the distance from the sample plane to the entry of the objective, d_2 is the distance from the exit of the objective to the detector plane and $\mathbf{M^N}$ is the RTM for the CRL.
$\mathbf{K}$ is therefore given in terms of $\mathbf{M^N}$ as:

$$\mathbf{K} = \begin{bmatrix} M_{11}^N + d_2 M_{21}^N & M_{12}^N + d_1(M_{11}^N + d_2 M_{21}^N) + d_2 M_{22}^N \\ M_{21}^N & d_1 M_{21}^N + M_{22}^N \end{bmatrix} \tag{30}$$

The imaging condition implies that:

$$\begin{bmatrix} 0 \\ \alpha_d \end{bmatrix} = \mathbf{K} \begin{bmatrix} 0 \\ \alpha_s \end{bmatrix} \tag{31}$$

i.e.:

$$K_{12} = 0 \tag{32}$$

Inserting this leads to:

$$f\varphi \sin(N\varphi) + d_1\left(\cos(N\varphi) - d_2\frac{\sin(N\varphi)}{f\varphi}\right) + d_2\cos(N\varphi) = 0 \tag{33}$$

This can rewritten as the *imaging condition* for a thick CRL:

$$\frac{1}{d_1} + \frac{1}{d_2} - \frac{1}{f_N} + \frac{f\varphi\tan(N\varphi)}{d_1 d_2} = 0 \tag{34}$$

Next, the magnification of the imaging system $\mathcal{M}$ (a positive number) is defined by:

$$K_{11} = \mathcal{M} = -\frac{y_d}{y_s} \tag{35}$$

We therefore have a set of two equations:

$$\begin{aligned} 0 &= M_{12}^N + d_1(M_{11}^N + d_2 M_{21}^N) + d_2 M_{22}^N \\ -\mathcal{M} &= M_{11}^N + d_2 M_{21}^N \end{aligned} \tag{36}$$

which can then be solved simultaneously to find expressions for d_1 and d_2:

$$d_1 = -\frac{M_{12}^N}{\mathcal{M}} + \frac{M_{11}^N M_{22}^N}{M_{21}^N \mathcal{M}} + \frac{M_{22}^N}{M_{21}^N} = \frac{f\sin(\varphi)}{\sin(N\varphi)}\left[\cos(N\varphi) + \frac{1}{\mathcal{M}}\right] = f_N\left[1 + \frac{1}{\mathcal{M}\cos(N\varphi)}\right] \tag{37}$$

$$d_2 = -\frac{\mathcal{M} - M_{11}^N}{M_{21}^N} = \frac{f\sin(\varphi)}{\sin(N\varphi)}[\cos(N\varphi) + \mathcal{M}] = f_N\left[1 + \frac{\mathcal{M}}{\cos(N\varphi)}\right] \tag{38}$$

Note that Eqs. 37 and 38 are identical for $\mathcal{M} = 1$. Moreover, d_1 and d_2 may never be smaller than f_N. For practical applications, the total sample-to-detector distance: $L = d_1 + d_2 + NT$ may be a fixed parameter, implying that we may want to rewrite the imaging condition as an expression in L and $\mathcal{M}$. Addition of these two equations then gives:

$$\frac{L - NT}{f\varphi}\sin(N\varphi) - 2\cos(N\varphi) = \mathcal{M} + \frac{1}{\mathcal{M}} \tag{39}$$

We can rewrite this using trigonometric identities (see Appendix):

$$\sqrt{4 + \left(\frac{L - NT}{f\varphi}\right)^2}\sin\left(N\varphi - \tan^{-1}(\frac{2f\varphi}{L - NT})\right) = \mathcal{M} + \frac{1}{\mathcal{M}} \tag{40}$$

Hence, for a given lenslet (i.e. f and φ), total distance L and magnification $\mathcal{M}$, we can determine the required number of lenses from:

$$N = \frac{1}{\varphi}\left(\sin^{-1}\left(\frac{\mathcal{M} + \frac{1}{\mathcal{M}}}{\sqrt{(\frac{L-NT}{f\varphi})^2 + 4}}\right) + \tan^{-1}\left(\frac{2f\varphi}{L - NT}\right)\right) \tag{41}$$

Due to the factor of N in the denominator, N should be determined by evaluating the equation twice *via*, e.g. the fixed-point method.

S7:Derivation of the acceptance functions for imaging systems

The expression for $\mathbf{K}$ (Eq. 30) provides the following expression for y_n in terms of the sample plane coordinates (y_s, α_s):

$$\begin{aligned} y_n &= \alpha_s\left[d_1\left(M_{11}^n - \frac{T}{2}M_{21}^n\right) + M_{12}^n - \frac{T}{2}M_{22}^n\right] + y_s\left(M_{11}^n - \frac{T}{2}M_{21}^n\right) \\ &= (d_1\alpha_s + y_s)\cos\left[\left(n - \frac{1}{2}\right)\varphi\right] + f\varphi\alpha_s\sin\left[\left(n - \frac{1}{2}\right)\varphi\right] \end{aligned} \tag{42}$$

The cumulative attenuation for the entire imaging system, U_N, can be derived in the same manner as for the CRL alone. Temporarily neglecting the physical aperture of the lenslet, this is can be written as:

$$U_N(y_s,\alpha_s) = \exp\left(-N\mu T_{\text{web}}\right) \times \exp\left[-(A_N^*\alpha_s^2 + B_N^* y_s\alpha_s + C_N^* y_s^2)\right] \tag{43}$$

where the coefficients A_N^*, B_N^* and C_N^* are geometrical sums which, using the identities in the Appendix, can be derived as follows:

$$\begin{aligned}
A_N^* &= \frac{\mu}{R}\sum_{n=1}^{N}\left[d_1\left(M_{11}^n - \frac{T}{2}M_{21}^n\right) + \left(M_{12}^n - \frac{T}{2}M_{22}^n\right)\right]^2 \\
&= \frac{\mu}{R}\sum_{n=1}^{N}\left[d_1(\cos(n\varphi) + \frac{\varphi}{2}\sin(n\varphi)) + f\varphi\sin(n\varphi) - \frac{T}{2}\cos(n\varphi)\right]^2 \\
&= \frac{\mu}{R}\left[d_1^2 + (f\varphi)^2\right]\sum_{n=1}^{N}\cos^2\left(\left(n-\frac{1}{2}\right)\varphi - \cot^{-1}\left(\frac{d_1}{f\varphi}\right)\right) \\
&= \frac{\mu}{2R}\left[d_1^2 + (f\varphi)^2\right]\left[N - 1 + \frac{1}{\varphi}\sin\left((N+1)\varphi\right)\cos\left((N-1)\varphi - 2\cot^{-1}\left(\frac{d_1}{f\varphi}\right)\right)\right]
\end{aligned} \tag{44}$$

$$\begin{aligned}
B_N^* &= \frac{2\mu}{R}\sum_{n=1}^{N}\left(M_{11}^n - \frac{T}{2}M_{21}^n\right)\left[d_1\left(M_{11}^n - \frac{T}{2}M_{21}^n\right) + \left(M_{12}^n - \frac{T}{2}M_{22}^n\right)\right] \\
&= \frac{2\mu}{R}\sum_{n=1}^{N}\left[d_1(\cos(n\varphi) + \frac{\varphi}{2}\sin(n\varphi)) + f\varphi\sin(n\varphi) - \frac{T}{2}\cos(n\varphi)\right]\left(\cos(n\varphi) + \frac{\varphi}{2}\sin(n\varphi)\right) \\
&= \frac{2\mu}{R}\left[\sum_{n=1}^{N} d_1\cos\left(\left(n-\frac{1}{2}\right)\varphi\right) + \sum_{n=1}^{N} f\varphi\sin\left(\left(n-\frac{1}{2}\right)\varphi\right)\cos\left(\left(n-\frac{1}{2}\right)\varphi\right)\right] \\
&= \frac{2\mu}{R}\left[\frac{d_1}{2}(N-1) + \sqrt{d_1^2 + (f\varphi)^2}\frac{\sin\left((N+1)\varphi\right)}{2\varphi}\cos\left((N-1)\varphi - \cot^{-1}\left(\frac{d_1}{f\varphi}\right)\right)\right]
\end{aligned} \tag{45}$$

$$\begin{aligned}
C_N^* &= \frac{\mu}{R}\sum_{n=1}^{N}\left(M_{11}^n - \frac{T}{2}M_{21}^n\right)^2 = C_N \\
&= \frac{N\mu}{2R}\left(1 + \text{sinc}(2N\varphi)\right)
\end{aligned} \tag{46}$$

The function $U_N(y_s,\alpha_s)$ is also a tilted 2D Gaussian. It can be described as the product of a constant term $\exp\left(-N\mu T_{\text{web}}\right)$ and two 1D Gaussian functions:

$$U_N(y_s,\alpha_s) = \exp\left(-N\mu T_{\text{web}}\right) \times \exp\left(\frac{-(\alpha_s - \gamma y_s)^2}{2\sigma_a^2}\right) \times \exp\left(\frac{-y_s^2}{2\sigma_v^2}\right) \tag{47}$$

These two functions are the *vignetting function*, which describes the reduction in brightness from the centre of the optical axis towards its periphery in terms of an RMS σ_v, and the *angular acceptance function*, which describes the angle over which the lens collects radiation emitted from a point y_s on the sample plane. The angular acceptance function has RMS σ_a and offset γ. Their individual expressions are derived from the coefficients A_N^*, B_N^* and C_N^* in the following subsections.

A. Angular acceptance

By inspection of Eq. 43, the angular acceptance function is a Gaussian with standard deviation σ_a given by:

$$\begin{aligned}
\sigma_a &= \sqrt{\frac{1}{2A_N^*}} \\
&= \sqrt{\frac{R}{\mu N[d_1^2 + (f\varphi)^2]}}\left[1 + \frac{1}{N} - \frac{1}{N\varphi}\sin\left((N+1)\varphi\right)\cos\left((N-1)\varphi + 2\tan^{-1}\left(\frac{d_1}{f\varphi}\right)\right)\right]^{-\frac{1}{2}}
\end{aligned} \tag{48}$$

The last term is a slowly varying function that asymptotically approaches 1 for $N\varphi \to 0$. The analytical expression for γ can similarly be expressed as:

$$\begin{aligned}\gamma &= \frac{B_N^*}{2A_N^*} \\ &= \frac{(f+2d_1N)\sqrt{d_1^2+(f\varphi)^2}\cos\left(2N\varphi+\tan^{-1}\left(\frac{d_1}{f\varphi}\right)\right)}{2[d_1f+N(d_1^2+f^2)]+\frac{1}{\varphi}[d_1^2+(f\varphi)^2]\sin(2N\varphi)}\end{aligned} \tag{49}$$

B. Vignetting

By inspection of Eq. 43, the RMS value σ_v is given by:

$$\begin{aligned}\sigma_v &= \sqrt{\frac{2A_N^*}{4A_N^*C_N^*-B_N^{*2}}} \\ &= \frac{\delta}{\mu\sigma_a}\left[(N\varphi)^2-\sin^2(N\varphi)\right]^{-\frac{1}{2}}\end{aligned} \tag{50}$$

S8: Derivation of spatial resolution

The spatial resolution, Δy_d, is the minimum distance two objects can be separated while still being 'distinguishable' by the imaging system. While the Rayleigh criterion[4] provides a simple and effective approximation for this, it is not applicable to systems with Gaussian pupil (i.e. attenuation) functions. Instead, we must first derive the point-spread function (PSF) of the system, then determine the minimum resolvable distance between two such PSFs.

In an ideal imaging configuration, the PSF is simply the Fourier transform of the pupil function, $P(y_0)$[4]:

$$PSF(y_s) = \mathcal{F}(P(y_0)) \tag{51}$$

where the pupil function is the acceptance of a ray emanating from the centre of the field-of-view, derived from Eq. 47:

$$P(y_0) = \exp\left(\frac{-y_0^2}{2\sigma_a^2d_1^2}\right)\times H\left(\frac{y_0}{Y_{\text{pup}}}\right) \tag{52}$$

Here, $Y_{\text{pup}} \equiv Y_{\text{phys}}/\sqrt{1+(f\varphi/d_1)^2}$. For a wavenumber $k = 2\pi/\lambda$ at x-ray wavelength λ, and ignoring constant prefactors, the point-spread intensity function at the source plane, $PSF(y_s)$, is then given by:

$$\begin{aligned}PSF(y_s) &= \left|\int_{-\infty}^{\infty} P(y_0)\exp\left[-i\frac{k}{d_1}y_0y_s\right]\mathrm{d}y_0\right|^2 \\ &= \exp\left(-k^2\sigma_a^2y_s^2\right)\times\left[\operatorname{erf}\left(\frac{Y_{\text{pup}}+ik\sigma_a^2y_s}{\sqrt{2}\sigma_a}\right)+\operatorname{erf}\left(\frac{Y_{\text{pup}}-ik\sigma_a^2y_s}{\sqrt{2}\sigma_a}\right)\right]^2\end{aligned} \tag{53}$$

The resolution can then be defined by the the separation distance between two PSFs corresponding to a contrast ratio of C (where C is small when the contrast is poor). Using Eq. 53, this can be determined by numerically solving:

$$\frac{PSF(0)+PSF(\Delta y_s)}{PSF(\frac{\Delta y_s}{2})+PSF(-\frac{\Delta y_s}{2})} = 1-C \tag{54}$$

In the case of absorption-limited (i.e. Gaussian) CRLs, this gives a function in terms of λ, σ_a and C:

$$\Delta y_d = \sqrt{0.06905-0.1019\log(1-C)}\frac{\lambda}{\sigma_a} \tag{55}$$

S9: Derivation of chromatic aberration

The refractive decrement δ is approximately inversely proportional to the square of the of the x-ray energy. Hence, compared to δ_0 at a nominal energy E_0, the actual δ at energy E will be:

$$\delta \approx \delta_0 \frac{E_0^2}{E^2} = \frac{\delta_0}{(1+\epsilon)^2} \tag{56}$$

Where ϵ is a (small) normalised energy perturbation such that $E/E_0 = 1 + \epsilon$. This energy dependence of δ means that the values for f and φ in the formalism are similarly chromatic. Their new values values are:

$$\varphi = \frac{\varphi_0}{1+\epsilon} \approx \varphi_0(1-\epsilon), \qquad f = f_0(1+\epsilon)^2 \approx f_0(1+2\epsilon) \tag{57}$$

A ray originating from the sample at $y_s = 0$ will strike the detector at y_d according to:

$$y_d = \alpha_s \left[(d_1 + d_2)\cos(N\varphi) + \left(f\varphi - \frac{d_1 d_2}{f\varphi} \right) \sin(N\varphi) \right] \tag{58}$$

If we are in an ideal imaging condition at $E = E_0$, then $y_{d,0} = y_s = 0$. However, if $E \neq E_0$ then the ray will strike the detector at $y_d \neq y_s = 0$. The dependence of y_d on ϵ is found by substituting the chromatic expressions for ϕ and f and Taylor expanding to first order in ϵ:

$$y_d = y_{d,0} + d_{\mathrm{ch}}\epsilon\alpha_s = d_{\mathrm{ch}}\epsilon\alpha_s \tag{59}$$

where:

$$d_{\mathrm{ch}} = \frac{N\varphi_0[d_1 d_2 - (f_0\varphi_0)^2]\cos(N\varphi_0) + [d_1 d_2 + (f_0\varphi_0)^2 + f_0 N\varphi_0^2(d_1+d_2)]\sin(N\varphi_0)}{f_0\varphi_0} \tag{60}$$

To calculate the intensity of a chromatic ray, we first consider that the incident ray will have an intensity at the sample I_ϵ defined by the chromatic distribution of the source. In many cases this is Gaussian with an RMS bandwidth σ_e:

$$I_\epsilon = I_0 \frac{1}{\sqrt{2\pi}\sigma_e} \exp\left(\frac{-\epsilon^2}{2\sigma_e^2} \right) \tag{61}$$

The ray will then be attenuated by the material in the lens as a function of its incident angle α_s. From the formalism we know that this is a Gaussian attenuation function with RMS width σ_a given by:

$$U(\alpha_s) = \exp\left(\frac{-\alpha_s^2}{2\sigma_a^2} \right) \tag{62}$$

Thus, the intensity of the ray at the detector position, I_d, will be:

$$I_d(\epsilon, \alpha_s) = I_\epsilon \times U(\alpha_s) = I_0 \frac{1}{\sqrt{2\pi}\sigma_e} \exp\left(\frac{-\epsilon^2}{2\sigma_e^2} \right) \times \exp\left(\frac{-\alpha_s^2}{2\sigma_a^2} \right) \tag{63}$$

From Eq. 58, we can calculate the initial angle of a ray given its ϵ and y_d as $\alpha_s = y_d/(d_{\mathrm{ch}}\epsilon)$. Substituting this into Eq. 63 then gives the spatio-chromatic intensity distribution at the detector:

$$I_d(\epsilon, y_d) = I_0 \frac{1}{\sqrt{2\pi}\sigma_e} \exp\left(\frac{-\epsilon^2}{2\sigma_e^2} \right) \times \exp\left[\frac{-y_d^2}{2(\sigma_a d_{ch}\epsilon)^2} \right] \tag{64}$$

Normalising y_d by the system magnification $\mathcal{M}$ then gives the perceived chromatic point-spread in the sample coordinates, y_s:

$$I_s(\epsilon, y_d) = I_0 \frac{1}{\sqrt{2\pi}\sigma_e} \exp\left(\frac{-\epsilon^2}{2\sigma_e^2} \right) \times \exp\left[\frac{-y_s^2}{2(\sigma_a d_{ch}\epsilon\mathcal{M})^2} \right] \tag{65}$$

So the chromatic point-spread function (PSF_{chr}) is then obtained by integrating I_d across ϵ:

$$\begin{aligned} PSF_{\mathrm{ch}} &= I_0 \frac{1}{\sqrt{2\pi}\sigma_e} \int_{-\infty}^{\infty} \exp\left(\frac{-\epsilon^2}{2\sigma_e^2} \right) \times \exp\left(\frac{-y_s^2}{2(\sigma_a d_{\mathrm{ch}}\mathcal{M}\epsilon)^2} \right) \mathrm{d}\epsilon \\ &= I_0 \exp\left(\frac{-|y_s|}{\sigma_e\sigma_a\mathcal{M}d_{\mathrm{ch}}} \right) \end{aligned} \tag{66}$$

Which is a Laplacian distribution with characteristic width:

$$\sigma_{\mathrm{ch}} = \sigma_a\sigma_e\mathcal{M}d_{\mathrm{ch}} \tag{67}$$

Appendix: Trigonometric Identities

Throughout this work, we will make use of the following identities:

$$a\cos(\theta) + b\sin(\theta) = \sqrt{a^2+b^2}\cos\left(\theta - \cot^{-1}\left(\frac{a}{b}\right)\right) = \sqrt{a^2+b^2}\sin\left(\theta + \tan^{-1}\left(\frac{a}{b}\right)\right) \tag{A.1}$$

And:

$$\sum_{n=1}^{N}\sin^2(n\theta+d) = \frac{1}{2}\left(N+1-\frac{\sin((N+1)\theta)}{\sin\theta}\cos(N\theta+2d)\right) \tag{A.2}$$

$$\sum_{n=1}^{N}\cos^2(n\theta+d) = \frac{1}{2}\left(N-1+\frac{\sin((N+1)\theta)}{\sin\theta}\cos(N\theta+2d)\right) \tag{A.3}$$

$$\sum_{n=1}^{N}\cos(n\theta+d)\sin(n\theta+d) = \frac{1}{2}\left(\frac{\sin((N+1)\theta)}{\sin\theta}\sin(N\theta+2d)\right) \tag{A.4}$$

Furthermore, we utilize that $\tan^{-1}(x) = \cot^{-1}\left(\frac{1}{x}\right) \approx x$ for $x \ll 1$
Finally we mention the following two identities

$$\sin(\cot^{-1}(x)) = \frac{1}{\sqrt{1+x^2}} \tag{A.5}$$

$$\cos(\cot^{-1}(x)) = \frac{x}{\sqrt{1+x^2}} \tag{A.6}$$

$$\tag{A.7}$$

[1] J. Als-Nielsen and D. McMorrow, *Elements of modern X-ray physics* (Wiley, 2010).
[2] B. Lengeler, C. Schroer, J. Tümmler, B. Benner, M. Richwin, A. Snigirev, I. Snigireva, and M. Drakopoulos, J. of Synchrotron Radiat. **6**, 1153 (1999), URL `http://dx.doi.org/10.1107/S0909049599009747`.
[3] A. Gerrard and J. M. Burch, *Introduction to matrix methods in optics* (Courier Corporation, 2012).
[4] M. Born and E. Wolf, *Principles of Optics* (Cambridge University Press, 1999), 7th ed.
